# Clinical Outcomes on Remote Monitoring Compliance in Patients With Implantable Cardioverter Defibrillator

**DOI:** 10.1002/joa3.70203

**Published:** 2025-10-08

**Authors:** Vern Hsen Tan, Hui Xin See Tow, Khi Yung Fong, Xuan Han Koh, Yue Wang, Ai Ling Him, Sherida Syed Hamid, Xiao Rong Joscelin Mok, Leng Leng Lee, Colin Yeo, Chi Keong Ching

**Affiliations:** ^1^ Department of Cardiology Changi General Hospital Singapore Singapore; ^2^ Yong Loo Lin School of Medicine National University of Singapore Singapore Singapore; ^3^ Duke‐NUS Medical School Singapore Singapore; ^4^ Singapore Health Services Singapore Singapore; ^5^ Department of Nursing Changi General Hospital Singapore Singapore; ^6^ Clinical Measurement Unit Changi General Hospital Singapore Singapore; ^7^ Department of Cardiology National Heart Centre Singapore Singapore Singapore

**Keywords:** cardiac resynchronization therapy, electrophysiology, implantable cardioverter‐defibrillator, remote monitoring

## Abstract

**Background:**

Remote monitoring (RM) is crucial in managing patients with cardiac implantable electronic devices (CIED), and has been reported to improve clinical outcomes. The study's objective is to investigate whether compliance to RM has affected patients' long‐term clinical outcomes.

**Methods:**

This was a prospective single‐center cohort study of consecutive patients on RM with an implantable cardioverter‐defibrillator or cardiac resynchronization therapy defibrillator followed up from 2018 to 2024. For analysis, patients were stratified according to whether they were compliant with RM. Compliance was defined as having ≥ 2 scheduled transmissions per year. Outcomes studied were the combined endpoints of all‐cause mortality or heart failure hospitalization.

**Results:**

We analyzed 207 patients, of whom 58 (28.1%) were compliant and 149 (71.9%) were non‐compliant. Baseline demographics were similar between both arms. We observed 6.9% (4/58) of compliant patients with all‐cause mortality, compared to 15.4% (23/149) of non‐compliant patients. Four percent (2/56) of compliant patients had a hospitalization for heart failure, compared to 12.2% (18/147) of non‐compliant patients. The Kaplan–Meier analysis suggested differences in the mortality and heart failure hospitalization survival function for compliant versus non‐compliant patients. Among 166 patients with available covariate data in the multivariable exponential accelerated failure time model, the time until mortality or heart failure hospitalization was longer for compliant compared to non‐compliant patients (acceleration factor 0.24, 95% CI 0.07 to 0.81; *p* = 0.022).

**Conclusion:**

RM compliance was associated with significantly lower combined end‐points of all‐cause mortality and heart failure hospitalizations, even though individual outcomes (mortality or HF hospitalization) did not reach statistical significance.

## Graphical abstract

Remote monitoring compliance was associated with significantly lower combined end‐points of all‐cause mortality and heart failure hospitalizations, even though individual outcomes (mortality or heart failure hospitalization) did not reach statistical significance.

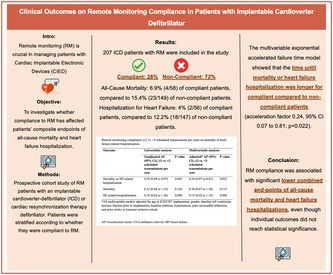

## 1 Introduction

Remote monitoring (RM) is an important part of managing patients with cardiac implantable electronic devices (CIEDs) and is regarded as the standard of care for these patients [1]. Recent guidelines from the European Society of Cardiology and the American Heart Association indicate that all patients with CIEDs should be offered RM as part of their management [1, 2]. CIEDs are increasingly being implanted in patients and include implantable cardioverter‐defibrillators (ICD) and cardiac resynchronization therapy‐defibrillators (CRT‐D) [3].

RM allows for manual or auto‐transmissions initiated by the patient's device if preset criteria are met or if patients trigger transmissions based on symptoms [4]. The data are transmitted wirelessly to a transceiver close to the patient or the patient's smart mobile device, then to the manufacturer's central repository using a wireless data network. Healthcare providers, such as physicians and cardiac physiologists, retrieve and review this data [4].

RM provides the benefits of frequent patient updates without their physical presence in clinics, and allows for fast response to patient and device events [5]. The increasing adoption of RM reflects its potential and benefits in enhancing clinical outcomes, such as reducing in‐clinic evaluations and reducing heart failure hospitalization and mortality [6, 7]. Multiple studies have demonstrated the safety and reliability of RM in the timely detection of arrhythmias and other cardiac complications, ultimately leading to improved management of cardiac conditions [8, 9, 10]. Furthermore, the continuity of monitoring is of paramount importance, as a study has shown that patients who adhere to RM and transmit data consistently are at substantially lower risk of death and hospital readmission [11].

However, despite the promising results observed in larger cohorts, data on RM compliance and its impact on long‐term clinical outcomes remain limited, particularly in the Asia‐Pacific region. This study aims to evaluate whether compliance with RM affects long‐term outcomes, specifically all‐cause mortality and heart failure hospitalizations, in a cohort of patients with ICDs and CRT‐Ds at a single institution in Singapore.

## 2 Methods

### 2.1 Study Design and Participants

This was a prospective, single‐center, observational cohort study of consecutive patients who were seen in our institution's CIED clinic from December 2018 to May 2024. Our institution is a public healthcare hospital serving a population of approximately 1 million on the eastern side of Singapore.

Patients implanted with an ICD or CRT‐D from any manufacturer (Abbott [previously known as St. Jude Medical], Biotronik, Boston Scientific, or Medtronic) who underwent follow‐up in our institution during the study period were included in the analysis. Devices were implanted by trained electrophysiologists. Device programming including tachycardia detection and therapy was programmed according to expert consensus statements in literature [12]. Patients had to have at least 3 months of follow‐up in clinic prior to enrolment into the study. Exclusion criteria included patients receiving non‐ICD devices such as cardiac resynchronization therapy‐pacemakers (CRT‐P), pacemakers, or implantable loop recorders; patients with unsuccessful device implantation; or patients who did not give consent to participate in the study.

In general, all patients implanted with an ICD or CRT‐D were offered bedside RM. Patients were followed up with in‐person visits at 2–3 months post‐implantation, then again at 6 months. Subsequent follow‐up intervals ranged from 1 to 2 years, depending on the electrophysiologist's assessment. For patients in the RM arm, all RM in this study involved in‐built monitoring technologies of the respective CIEDs. These include the recording of device therapy (anti‐tachycardia pacing [ATP] or shocks) and inciting events such as ventricular tachycardia or ventricular fibrillation, changes in lead impedance, sensing or capture thresholds, atrial arrhythmia burden, ventricular pacing percentage, and battery reserve. These alerts are wirelessly transmitted automatically to the respective manufacturer's cloud server. The cardiac physiologist reviewed the alerts during working hours, and notable events will be reviewed by the electrophysiologist, triggering an earlier clinic visit. Patient‐initiated transmission was measured by the frequency of patient‐initiated transmission and median utilization of patient‐initiated transmission per patient [13].

### 2.2 Definitions

Patients were categorized according to whether they were compliant with RM. Compliance with RM was defined as having ≥ 2 scheduled transmissions per year based on existing publication [14]. Scheduled transmissions are defined as transmissions that occur at regular intervals [14, 15]. At our institution, scheduled transmissions occur once every 6 months. Unscheduled transmissions are defined as transmissions that occur due to programmable alert conditions or if abnormal criteria were detected [4, 15]. Unscheduled transmissions can be further classified into valid and invalid transmissions [15]. Valid transmissions are alerts requiring urgent or non‐urgent clinical response [16], which include ventricular tachycardia, ventricular fibrillation (VT/VF), battery issues, device issues, ventricular intrinsic amplitude out of range, atrial intrinsic amplitude out of range, non‐sustained tachycardia, ventricular sensing episodes (for CRTD), ventricular pacing < 90% (for CRTD), crossing of the left or right ventricular threshold, possible fluid accumulation, crossing of the Heart Failure Index, atrial tachycardia or AF in a patient without AF [16]. All transmissions indicating crossing of the Heart Failure Index were deemed valid and considered unscheduled. Invalid transmissions are alerts that do not require clinical response and include false positive AF transmissions in a known AF patient, noise, low patient activity, or no event [16]. Patient‐initiated transmissions are defined as transmissions triggered by patients based on their symptoms [4].

### 2.3 Outcomes

The primary outcome for this study was a combination of all‐cause mortality or heart failure hospitalization during follow‐up. The secondary outcomes were all‐cause mortality, hospitalization for heart failure, as well as CIED‐delivered therapies. These included ATP, shocks, and electrical storm. These therapies were further categorized as appropriate or inappropriate. All clinical and CIED events during this period were retrieved from our institution's electronic medical records. Institutional review board (IRB) approval was received for the conduct of this study (2019/2415) and all patients provided written informed consent for the use of anonymized data.

### 2.4 Statistical Analysis

The reporting of study results was in accordance with STROBE guidelines [17]. Descriptive statistics of baseline patient demographic and clinical characteristics overall and by the number of scheduled transmissions per year will be reported as number and percentage for categorical data, mean ± standard deviation (SD) for normally distributed data, and median and interquartile range (IQR) for non‐normally distributed data. To examine if there were differences between patients with ≥ 2 (i.e., compliant) versus < 2 (i.e., non‐compliant) scheduled transmissions per year, the Mann–Whitney *U* test was used for continuous characteristics while the chi‐squared test or Fisher's exact test was used for categorical characteristics. We generated plots of the Kaplan–Meier estimator for the mortality and heart failure (HF) hospitalization‐free probability against follow‐up time. The log‐rank test assessed if there were differences in the survival distributions of compliant versus non‐compliant patients.

In the main analysis, we first assessed the plausibility of the proportional hazards assumption graphically using plots of scaled Schoenfeld residuals against time, and quantitatively via a global test. As there was evidence of nonproportional hazards, we selected among accelerated failure time (AFT) models by considering the plot of log(−log[survival]) vs. log(time), Akaike information criterion (AIC) and Bayesian information criterion (BIC) values, as well as a priori subject knowledge about the hazard shape. We adopted the exponential AFT model as it had the lowest AIC and BIC among competing models, and based on the expected shape of the baseline hazard [18]. All multivariable models adjusted for age at ICD/CRT implantation, gender, baseline left ventricular ejection fraction prior to implantation, baseline diabetes, hypertension, prior myocardial infarction, and prior stroke or transient ischemic attack.

In the sensitivity analysis, we assessed the robustness of the main analysis results to alternative survival distribution assumptions. Differences in the time until the event between compliant versus non‐compliant patients were reported as acceleration factors with 95% confidence intervals (CIs). We also assessed if the effect of remote monitoring compliance on clinical outcomes was confounded by a year of ICD/CRT implantation (in or after 2016 vs. before 2016) by including this variable as a covariate in the multivariable exponential AFT model.

To explore the temporal trend in remote monitoring compliance, we plotted the percentage of patients with ≥ 2 scheduled transmissions per year and the median number of scheduled transmissions per year against the year of ICD/CRT implantation. Binomial exact 95% CIs were plotted on the same graph. To identify the baseline factors associated with non‐compliance (i.e., < 2 scheduled transmissions per year), we performed univariable and multivariable logistic regression with robust standard errors. Potential explanatory factors were selected a priori based on the literature and expert clinical opinion to avoid spurious findings. The Box–Tidwell test was used to detect evidence of non‐linearity between the natural log odds of non‐compliance and continuous covariates. Statistical tests were two‐sided with a 0.05 significance level. All statistical analyses were conducted using Stata 18 (College Station, TX: StataCorp LLC).

## 3 Results

### 3.1 Study Population

**FIGURE 1:**
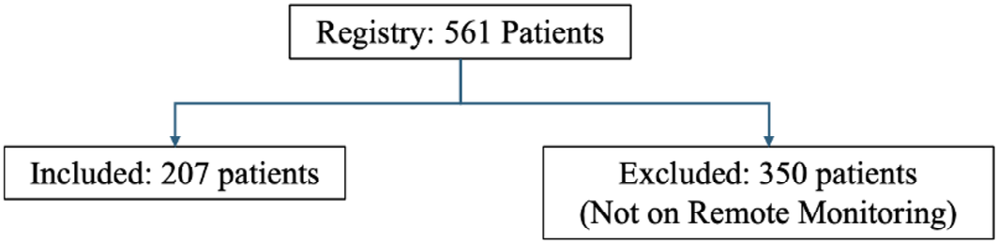
Flow Diagram of Included Patients.

Our registry included 561 patients, but 350 patients were excluded as they were not on remote monitoring, and 4 patients had CRT‐Pacemaker (Figure 1). Overall, we retrieved data from 207 patients with ICD or CRT‐D with RM, which were included in the analysis. Among the 207 patients included, 149 (71.9%) patients were non‐compliant (< 2 scheduled transmissions per year) and 58 (28.1%) patients were compliant (≥ 2 scheduled transmissions per year). The median age was 62 years (IQR 54–69), 82% (170/207) of patients were male, and the median follow‐up time was 6.6 years (IQR 3.9–9.5) (Table 1). Among the 149 non‐compliant patients, the median age was 61 years (IQR 52–69), 80.5% (120/149) were male, the median follow‐up time was 7.1 years (IQR 4.1–10.2), and the median no. of scheduled transmissions per year was 1.1 (IQR 0.5–1.7). Among the 58 compliant patients, the median age was 64 years (IQR 58–70), 86.2% (50/58) were male, the median follow‐up time was 5.9 years (IQR 3.3–7.7), and the median no. of scheduled transmissions per year was 2.2 (IQR 2.1–2.4). The majority of patients were on beta‐blockers, angiotensin‐converting enzyme inhibitors or angiotensin receptor blockers, statins, and antiplatelets.

**TABLE 1:** Baseline characteristics of patients overall and by the number of scheduled transmissions per year.

Characteristic	All (*n* = 207)	< 2 scheduled transmissions per year (*n* = 149)	≥ 2 scheduled transmissions per year (*n* = 58)	*p*
Age at ICD/CRT implant, median (IQR)	62 (54–69)	61 (52–69)	64 (58–70)	0.033
Male, *n* (%)	170 (82.1)	120 (80.5)	50 (86.2)	0.339
Pre‐implant LVEF, median (IQR)	25 (20–30)	25 (16–30)	25 (20–30)	0.804
Hypertension, *n* (%)	116 (57.1)	86 (58.9)	30 (52.6)	0.417
Heart disease, *n* (%)				
Ischemic CMP	143 (71.9)	98 (69.0)	45 (79.0)	0.556
Non‐ischemic CMP	31 (15.6)	23 (16.2)	8 (14.0)	
Brugada syndrome	4 (2.0)	4 (2.8)	0 (0)	
HCM	6 (3.0)	6 (4.2)	0 (0)	
Idiopathic VF	1 (0.5)	1 (0.7)	0 (0)	
LQTS	6 (3.0)	5 (3.5)	1 (1.8)	
Others/unknown	8 (4.0)	5 (3.5)	3 (5.3)	
Atrial fibrillation, *n* (%)	48 (23.8)	37 (25.3)	11 (19.6)	0.394
Myocardial infarction, *n* (%)	137 (69.9)	94 (66.7)	43 (78.2)	0.114
Previous revascularization, *n* (%)				
None	78 (39.2)	60 (42.0)	18 (32.1)	0.325
CABG	18 (9.1)	14 (9.8)	4 (7.1)	
PCI	92 (46.2)	63 (44.1)	29 (51.8)	
PCI and CABG	11 (5.5)	6 (4.2)	5 (8.9)	
Stroke/TIA, *n* (%)	25 (12.5)	18 (12.4)	7 (12.7)	0.952
Peripheral vascular disease, *n* (%)	7 (3.5)	7 (4.8)	0 (0)	0.194
Medication, *n* (%)				
Beta‐blockers	155 (76.0)	110 (74.8)	45 (79.0)	0.537
ACEI/ARB	129 (63.2)	93 (63.3)	36 (63.2)	0.989
CCB (dihydropyridine)	18 (8.8)	11 (7.5)	7 (12.3)	0.278
Loop diuretics	87 (42.7)	63 (42.9)	24 (42.1)	0.922
Potassium‐sparing diuretics	91 (44.8)	61 (41.8)	30 (52.6)	0.162
Statins	169 (82.8)	119 (81.0)	50 (87.7)	0.250
Hydralazine	3 (1.5)	0 (0)	3 (5.4)	0.020
Amiodarone	10 (4.9)	9 (6.1)	1 (1.8)	0.290
Antiplatelets	133 (65.2)	91 (61.9)	42 (73.7)	0.113
Warfarin	17 (8.3)	15 (10.2)	2 (3.5)	0.161
NOAC	20 (9.9)	15 (10.3)	5 (8.8)	0.747
Digoxin	19 (9.3)	16 (10.9)	3 (5.3)	0.215
Ivabradine	14 (6.9)	11 (7.5)	3 (5.3)	0.761
Sacubitril‐valsartan	35 (19.2)	22 (17.1)	13 (24.5)	0.245
SGLT2i	38 (20.9)	26 (20.2)	12 (22.6)	0.708
Device configuration, *n* (%)				
CRT‐D	31 (15.0)	23 (15.4)	8 (13.8)	0.062
DDD‐ICD	27 (13.0)	22 (14.8)	5 (8.6)	
VVI‐ICD	138 (66.7)	93 (62.4)	45 (77.6)	
SICD	11 (5.3)	11 (7.4)	0 (0)	
Manufacturer, *n* (%)				
Biotronik	48 (23.2)	19 (12.8)	29 (50)	< 0.001
Boston Scientific	33 (15.9)	31 (20.8)	2 (3.5)	
Medtronic	39 (18.8)	24 (16.1)	15 (25.9)	
Abbott (previously St. Jude Medical)	87 (42.0)	75 (50.3)	12 (20.7)	
Secondary prevention indication, *n* (%)	58 (28.6)	45 (30.6)	13 (23.2)	0.297

Abbreviations: ACEI = angiotensin‐converting enzyme inhibitor; ARB = angiotensin receptor blocker; ARVC = arrhythmogenic right ventricular cardiomyopathy; CABG = coronary artery bypass graft; CCB = calcium channel blocker; CMP = cardiomyopathy; CRT‐D = cardiac resynchronization therapy‐defibrillator; DCM = dilated cardiomyopathy; HCM = hypertrophic cardiomyopathy; ICD = implantable cardioverter‐defibrillator; IQR = interquartile range; LQTS = long QT syndrome; LVEF = left ventricular ejection fraction; NOAC = novel oral anticoagulant; PCI = percutaneous coronary intervention; SGLT2i = sodium‐glucose cotransporter‐2 inhibitor; SICD = subcutaneous implantable cardioverter‐defibrillator; TIA = transient ischemic attack; VF = ventricular fibrillation.

### 3.2 Transmissions

There was a higher median total number of transmissions among compliant compared to non‐compliant patients per patient per year (3.8 [2.6–5.6] versus 2.3 [1.3–4.1], *p* < 0.001) (Table 2). There were higher median scheduled transmissions per patient per year (2.2 [2.1–2.4] versus 1.1 [0.5–1.7]) and similar median unscheduled transmissions per patient per year among compliant compared to non‐compliant patients (1.3 [0.2–2.8] versus 1.0 [0.4–3.1], *p* = 0.924). There was a significantly lower median number of patient‐initiated transmissions per patient per year between compliant and non‐compliant patients (0 [0–0.4] versus 0.3 [0–0.7], *p* < 0.001). There were lower invalid transmissions among compliant compared to non‐compliant patients (0 [0–0.9] versus 0.5 [0–2.3], *p* = 0.006). There were no significant differences in the number of valid transmissions between compliant and non‐compliant groups.

**TABLE 2:** Remote monitoring variables and clinical outcomes of patients overall and by the number of scheduled transmissions.

Characteristic	All (*n* = 207)	< 2 scheduled transmissions per year (*n* = 149)	≥ 2 scheduled transmissions per year (*n* = 58)	*p*
Follow‐up time in years, median (IQR)	6.6 (3.9–9.5)	7.1 (4.1–10.2)	5.9 (3.3–7.7)	0.012
Total no. of transmissions per year, median (IQR)	2.7 (1.8–4.9)	2.3 (1.3–4.1)	3.8 (2.6–5.6)	< 0.001
Patient initiated	0.2 (0–0.6)	0.3 (0–0.7)	0 (0–0.4)	< 0.001
Scheduled	1.5 (0.7–2.1)	1.1 (0.5–1.7)	2.2 (2.1–2.4)	—
Unscheduled	1.2 (0.3–3.1)	1.0 (0.4–3.1)	1.3 (0.2–2.8)	0.924
Valid	0.2 (0–0.9)	0.1 (0–0.7)	0.4 (0–1.2)	0.072
Invalid	0.3 (0–1.7)	0.5 (0–2.3)	0 (0–0.9)	0.006
All‐cause mortality, *n* (%)	27 (13.0)	23 (15.4)	4 (6.9)	0.101
Appropriate ATP, *n* (%)	35 (18.3)	25 (18.4)	10 (18.2)	0.974
No. per patient, median (IQR)	1 (1–2)	1 (1–3)	1 (1–1)	0.643
Inappropriate ATP, *n* (%)	16 (7.7)	11 (7.4)	5 (8.6)	0.775
No. per patient, median (IQR)	3 (1–7)	4 (1–7.5)	2 (1–3)	0.590
Received shock, *n* (%)	49 (24.5)	39 (27.1)	10 (17.9)	0.173
Appropriate shock	33 (22)	25 (22.9)	8 (19.5)	0.652
No. per patient, median (IQR)	1 (1–3)	1 (1–3)	1 (1–5)	1.000
Inappropriate shock	18 (12.4)	15 (14.2)	3 (7.7)	0.400
No. per patient, median (IQR)	2 (1–3.5)	2 (1–4)	2 (2–3)	0.945
Reason for inappropriate shock, *n* (%)				
Atrial fibrillation/atrial tachycardia	11 (68.8)	9 (69.2)	2 (66.7)	1.000
SVT	1 (6.3)	1 (7.7)	0 (0)	
Lead fracture	1 (6.3)	1 (7.7)	0 (0)	
Others	3 (18.8)	2 (15.4)	1 (33.3)	
Appropriate storm, *n* (%)	1 (0.7)	0 (0)	1 (2.6)	0.271
Inappropriate storm, *n* (%)	0 (0)	0 (0)	0 (0)	—
Hospitalization for heart failure within 5 years post ICD/CRT implant, *n* (%)	20 (9.9)	18 (12.2)	2 (3.6)	0.064
Pulse generator change, *n* (%)	62 (36.7)	57 (47.1)	5 (11.4)	< 0.001
Normal end‐of‐life, *n* (%)	50 (86.2)	46 (86.8)	4 (80)	0.538
Duration from implant to pulse generator change in years, median (IQR)	7.7 (5.9–8.7)	7.7 (6.2–8.7)	5.3 (4.8–8.0)	0.240

Abbreviations: ATP = antitachycardia pacing; CRT = cardiac resynchronization therapy; ICD = implantable cardioverter‐defibrillator; IQR = interquartile range; SVT = supraventricular tachycardia.

### 3.3 Outcomes

#### 3.3.1 Remote Monitoring Compliance on Primary Outcomes

**FIGURE 2:**
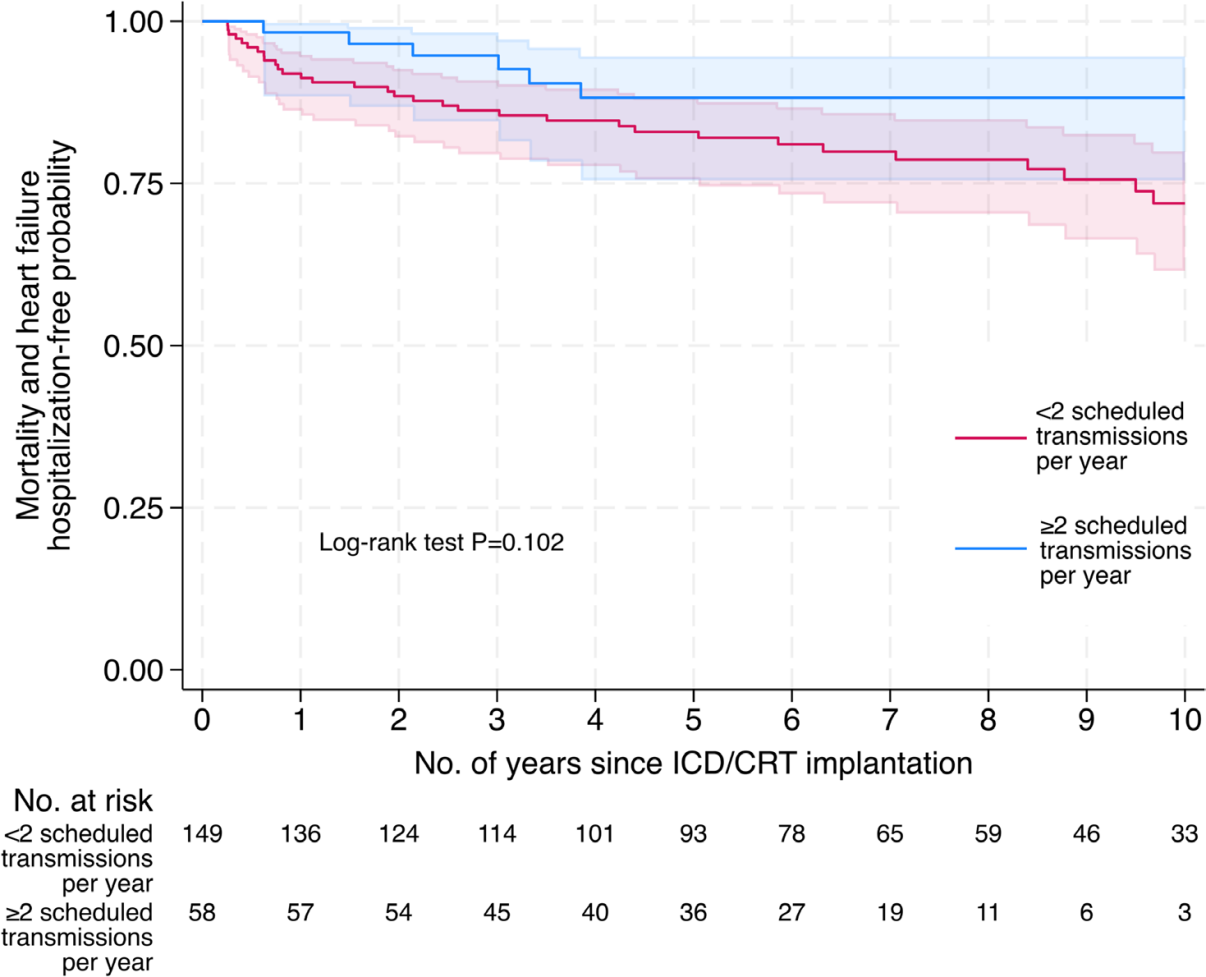
Kaplan–Meier estimates of primary outcome, by the number of scheduled transmissions per year (≥ 2 vs. < 2). CRT = cardiac resynchronization therapy; ICD = implantable cardioverter defibrillator.

We observed 6.9% (4/58) of compliant patients with all‐cause mortality, compared to 15.4% (23/149) of non‐compliant patients. Four percent (2/56) of compliant patients had a hospitalization for heart failure, compared to 12.2% (18/147) of non‐compliant patients. The Kaplan–Meier analysis suggested differences in the mortality and heart failure hospitalization survival function for compliant versus non‐compliant patients (Figure 2).

Among 166 patients with available covariate data in the multivariable exponential AFT model, the time until mortality or heart failure hospitalization was longer for compliant patients compared to non‐compliant patients (acceleration factor 0.24, 95% CI 0.07 to 0.81; *p* = 0.022; Table 3).

**TABLE 3:** Remote monitoring compliance (≥ 2 vs. < 2 scheduled transmissions per year) on mortality or heart failure‐related hospitalization.

Outcome	Univariable analysis		Multivariable analysis	
	Unadjusted AF (95% CI), ≥ 2 vs. < 2 scheduled transmissions per year	*p*	Adjusted* AF (95% CI), ≥ 2 vs. < 2 scheduled transmissions per year	*p*
Mortality or HF‐related hospitalization	0.29 (0.09 to 0.97)	0.044	0.24 (0.07 to 0.81)	0.022
Mortality	0.32 (0.08 to 1.35)	0.120	0.30 (0.07 to 1.34)	0.115
HF‐related hospitalization	0.18 (0.02 to 1.36)	0.096	0.15 (0.02 to 1.15)	0.068

Abbreviations: AF = acceleration factor; CI = confidence interval; HF = heart failure.

* All multivariable models adjusted for age at ICD/CRT implantation, gender, baseline left ventricular ejection fraction prior to implantation, baseline diabetes, hypertension, prior myocardial infarction, and prior stroke or transient ischemic attack.

#### 3.3.2 Remote Monitoring Compliance on Secondary Outcomes

**FIGURE 3:**
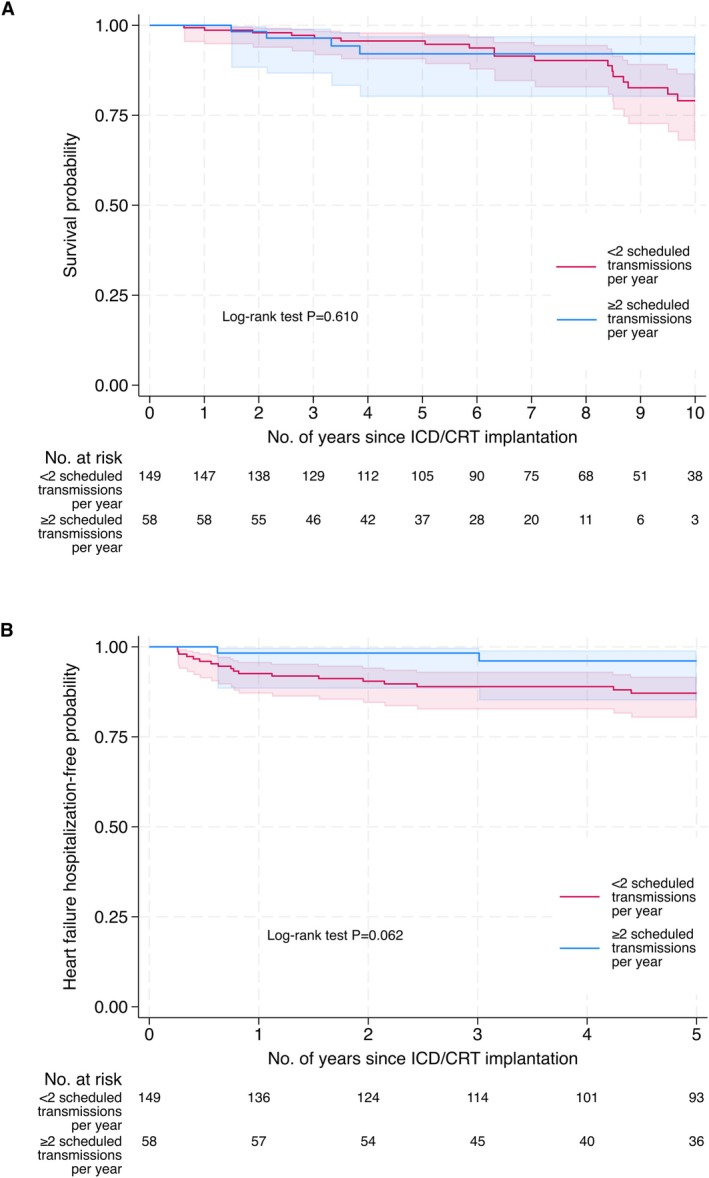
Kaplan–Meier estimates of secondary outcomes, by the number of scheduled transmissions per year (≥ 2 vs. < 2). (A) Mortality. (B) Hospitalization for heart failure. CRT = cardiac resynchronization therapy; ICD = implantable cardioverter defibrillator.

The Kaplan–Meier analysis suggested that the survival probability may differ between compliant and non‐compliant patients in a time‐dependent manner (Figure 3A), and that there may be a difference in the probability of remaining free from heart failure hospitalization (Figure 3B). Among 166 patients with available covariate data in the multivariable exponential AFT model, the time until mortality (acceleration factor 0.30, 95% CI 0.07 to 1.34; *p* = 0.115; Table 3) and heart failure hospitalization (acceleration factor 0.15, 95% CI 0.02 to 1.87; *p* = 0.068; Table 3) may be longer for compliant patients compared to non‐compliant patients. Although there was a significant reduction in combined mortality and heart failure hospitalization among compliant patients, secondary outcomes, including mortality or heart failure hospitalization alone, did not reach statistical significance. All multivariable models adjusted for age at ICD/CRT implantation, gender, baseline left ventricular ejection fraction prior to implantation, baseline diabetes, hypertension, prior myocardial infarction, and prior stroke or transient ischemic attack.

Outcomes and event rates at the end of follow‐up are listed in Table 2. There was no significant difference in device therapy (appropriate and inappropriate shocks and electrical storms) between compliant and non‐compliant patients. Among the episodes of inappropriate shock, atrial fibrillation (AF) was also the predominant cause (66.7% in compliant patients and 69.2% in non‐compliant patients). Electrical storm only occurred in 1 patient in the RM arm and was deemed appropriate.

#### 3.3.3 Sensitivity Analysis

Sensitivity analyses using multivariable Cox proportional hazards regression, log‐normal AFT, log‐logistic AFT, and Weibull AFT models showed consistent results for the primary and secondary outcomes (Table S1). We also assessed if the effect of remote monitoring compliance on clinical outcomes was confounded by the year of ICD/CRT implantation. After additionally adjusting for the year of ICD/CRT implantation, the exponential AFT model showed consistent results for the primary and secondary outcomes (Table S2).

#### 3.3.4 Baseline Factors Associated With Remote Monitoring Non‐Compliance

**FIGURE 4:**
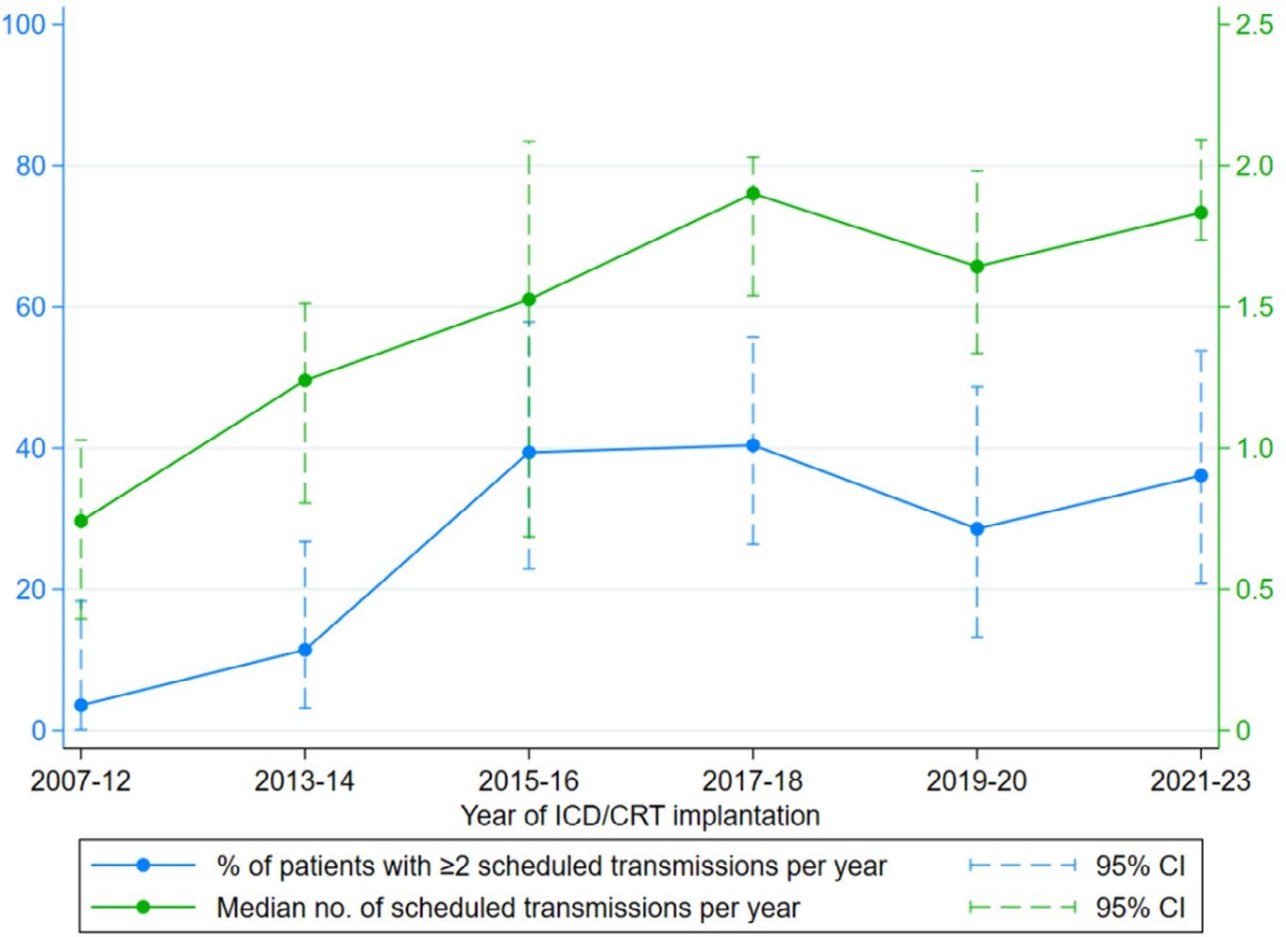
Remote monitoring compliance by year of ICD/CRT implantation. CI = confidence interval; CRT = cardiac resynchronization therapy; ICD = implantable cardioverter defibrillator.

In Figure 4, there was a noticeable increase in compliance among patients with an ICD/CRT implantation in or after 2016 compared to those with an implantation before 2016. As the Box–Tidwell showed evidence of non‐linearity between the natural log odds of non‐compliance and year of implantation, we dichotomized the year of implantation variable to after versus before 2016.

In Table 4, among 197 patients with available covariate data, multivariable logistic regression showed that compared to patients with an ICD/CRT implantation before 2016, those with an implantation in or after 2016 had 78% lower odds of non‐compliance (OR 0.22, 95% CI 0.10 to 0.51; *p* < 0.001). While older age at ICD/CRT implantation was associated with lower odds of non‐compliance in the univariable logistic regression analysis, this association did not persist after adjusting for potential confounders (Table 4).

**TABLE 4:** Baseline factors associated with remote monitoring non‐compliance (< 2 scheduled transmissions per year).

Baseline factor	Univariable logistic regression		Multivariable logistic regression	
	Unadjusted OR (95% CI)	*p*	Adjusted OR (95% CI)	*p*
Year of ICD/CRT implant				
Before 2016	1.00 (ref.)	—	1.00 (ref.)	—
In or after 2016	0.20 (0.09 to 0.45)	< 0.001	0.22 (0.10 to 0.51)	< 0.001
10‐year increase in age at ICD/CRT implant	0.74 (0.56 to 0.97)	0.027	0.83 (0.63 to 1.10)	0.196
Female (vs. male)	1.66 (0.68 to 4.06)	0.269	1.43 (0.55 to 3.72)	0.463
Atrial fibrillation (yes vs. no)	1.32 (0.62 to 2.83)	0.476	1.51 (0.69 to 3.34)	0.305
Secondary (vs. primary) prevention indication	1.35 (0.66 to 2.78)	0.410	1.01 (0.46 to 2.24)	0.982

Abbreviations: CI = confidence interval; CRT‐D = cardiac resynchronization therapy‐defibrillator; DDD = dual chamber; HF = heart failure; ICD = implantable cardioverter‐defibrillator; OR = odds ratio; SICD = subcutaneous implantable cardioverter‐defibrillator.

## 4 Discussion

Our study demonstrated that ICD/CRT‐D patients' RM and good compliance have significantly improved long‐term clinical outcomes compared with non‐compliant patients, particularly lower combined endpoints of mortality and heart failure hospitalization, although individual outcomes in mortality or heart failure hospitalization did not reach statistical significance. However, the compliance in our study was low (28.0%). Although other studies have also shown the clinical benefit of RM, our study focuses on an Asian cohort and demonstrates this benefit among a multi‐ethnic population [3, 6].

These findings align with existing studies that RM can effectively improve patients' survival and enable management of potential complications [8, 10]. This contrasts with studies that show no significant mortality benefit in RM [19, 20]. Effective RM utilization allows for the timely detection of arrhythmias, fluid overload, decompensation, or progressively worsening heart failure and device issues, and prompt interventions by healthcare providers to provide management or further investigations [8]. Identifying these symptoms early could allow for prompt management, such as medication optimization, hence preventing heart failure hospitalization.

Our study showed that the majority of patients with RM were non‐compliant (71.9%). This is in contrast with existing publication by Rosenfeld et al. in which non‐compliance with RM was 21%. However, our follow‐up (6.6 years) was longer compared to the Rosenfeld et al. study (14 months). Some predictors of non‐compliance in our study were found to be implantation of ICD/CRT before 2016 and older age of participants, although only for the univariable logistic regression analysis. Compliance was likely to have increased for participants who had implantation of ICD/CRT post‐2016, as mandatory remote monitoring was introduced to the institution from 2016 onwards. Additionally, while older age was associated with lower odds of non‐compliance in the univariable logistic regression analysis, this difference does not seem to be significant in the multivariable analysis. This observation could likely be due to our small sample size. Educational efforts can be directed to older patients who may not be as technologically savvy and allow them to understand the benefits of using RM [14].

Our results showed that compliant patients with RM had a higher number of total transmissions than non‐compliant patients. The higher number of device transmissions among compliant patients could be due to patients' higher motivation in managing their health. Adherence to RM may facilitate better device management and follow‐up, leading to improved maintenance of the device. This could explain the lower patient‐initiated transmissions and invalid transmissions among compliant compared to non‐compliant participants in our findings. Additionally, although the difference in the number of unscheduled transmissions was not statistically significant, the higher number of unscheduled transmissions among compliant patients compared to non‐compliant patients suggested that compliant patients may receive early or timely intervention, potentially contributing to reduced hospitalizations or mortality.

To improve patients' compliance further, besides education and increase awareness of the role and importance of RM, smartphone‐based RM was introduced. The utilization of smartphone‐based RM has been demonstrated to further improve compliance and connectivity compared to bedside RM in non‐randomized trials [13, 21]. These findings were observed in both patients with pacemakers and defibrillators. A benefit of smartphone‐based RM includes the availability of a patient‐facing interface, which enables patients to engage in their own management actively. Patients are able to look at device data, trends regarding their condition, and initiate transmissions at their discretion [21]. Given that only 28% of patients were compliant with semi‐annual transmission, strategies to enhance adherence are critical. Evidence suggests that automated reminders (e.g., via SMS or phone) can significantly improve adherence in cardiovascular populations [22]. Remote monitoring programs that streamline device follow‐ups and integrate patient education and technical support also show promise in increasing utilization and reducing missed transmissions [23, 24]. Future work should explore combining these interventions, tailoring them to address technological literacy, psychosocial barriers, and ensuring ease of use, possibly via mobile health platforms.

From the operational perspective, healthcare teams involved in RM often have to assess a large volume of RM transmissions and alerts, and determine which require clinical action [16]. For instance, unscheduled transmissions might require follow‐up clinical management, or even hospital visits. This could be so as unscheduled transmissions could be due to patient or device complications, such as arrhythmia episodes or lead‐related issues. Unscheduled transmissions can be classified into valid and invalid transmissions, and our results indicated that invalid transmissions were almost negligible in both compliant and non‐compliant RM patients. As there is a significant staff burden in looking through the large volume of transmissions and alerts, interventions to identify valid or true positive transmissions requiring further assessment can help to improve this workload [16]. Additionally, interventions to merge all alerts from several manufacturers into one platform can also reduce the workload for healthcare staff [16]. Introducing artificial intelligence tools, such as RM alerts, to filter out essential alerts helps reduce notification workload, as demonstrated in a pilot study [16].

### 4.1 Limitations

While our study highlighted that RM compliance improves clinical outcomes, it is important to acknowledge certain limitations of our study. Our single‐centre design may limit the generalizability of the findings, particularly in diverse populations with varying healthcare access and infrastructure. We were unable to quantify heart failure hospitalizations of our patients outside our healthcare cluster, leading to underestimation. The relatively smaller size of the compliant group may influence the statistical strength of some outcomes and the generalizability of our findings. A lower compliance rate can introduce selection bias, as compliant patients may have different baseline characteristics compared to non‐compliant patients, such as health‐seeking behavior, digital literacy, technological access, psychosocial factors, and socioeconomic status. These differences could contribute to the observed differences in outcomes and limit the generalizability of our findings. Future studies are needed to explore strategies to enhance compliance and confirm these findings in more representative cohorts. Our RM compliance definition reflects current practice standards rather than current or future technological capabilities (e.g., smartphone‐based transmission). When we acknowledge that this threshold may not perfectly reflect evolving RM practices, it provides a pragmatic and standardized benchmark that is comparable to prior studies. It should be noted that unscheduled transmissions, such as those prompted by alerts or patient‐reported symptoms, were not included in our compliance definition. While these events differ from scheduled transmissions, they may also reflect an important aspect of patient engagement and warrant consideration in future studies. Our study did not quantify the reasons for non‐compliance with RM, such as consent withdrawal or financial costs of RM. Understanding barriers to patient compliance, such as technological challenges, patient education, or psychosocial factors can help improve interventions to enhance patient compliance [13, 25]. Our study did not identify the median time taken from RM alerts to alerts being reviewed by physicians or cardiac physiologists. Additionally, although baseline characteristics were well matched between compliant and non‐compliant patients, other residual confounders could have led to the lower mortality and heart failure hospitalization in compliant patients. Providing patient‐centric approaches catering to individual circumstances and improving patient education could improve compliance [25]. This can be further explored in future studies to improve compliance and ultimately enhance clinical outcomes. Another limitation is that patient satisfaction was not evaluated in this study. Patient acceptance of RM is variable, as some studies showed no difference in quality of life [26], while others have shown a high patient satisfaction rate with RM [27].

## 5 Conclusions

In this single‐centre study of 207 patients with an ICD or CRT‐D, compliance to bedside RM was associated with significantly lower combined endpoints of mortality and heart failure hospitalizations. However, secondary outcomes (mortality or HF hospitalization alone) did not reach statistical significance. This suggests that healthcare teams should focus efforts on encouraging compliance in order to maximize the known benefits of RM. On the other hand, healthcare teams should monitor non‐compliant patients more closely as they are at risk of poorer clinical outcomes and might have underlying reasons for non‐compliance, such as unfamiliarity with technology. As healthcare technology continues to evolve, integrating RM into managing patients with CIED will enhance their quality of care and provide better clinical outcomes. Nevertheless, future research should focus on broader populations and identify factors and effective strategies to improve compliance towards RM.

## Ethics Statement

The institutional committee (Domain Specific Review Board) has approved this study.

## Consent

Informed consent was taken from patients.

## Conflicts of Interest

The authors declare no conflicts of interest.

## Supporting information


**Data S1:** joa370203‐sup‐0001‐Supinfo.docx.

## Data Availability

The data that support the findings of this study are available on request from the corresponding author. The data are not publicly available due to privacy or ethical restrictions.
