# Supplementary material for: Clinical Outcomes on Remote Monitoring Compliance in Patients With Implantable Cardioverter Defibrillator
Source: J Arrhythm. 2025 Oct 8;41(5):e70203. doi: 10.1002/joa3.70203 (PMC12507724; doi:10.1002/joa3.70203)
Supplement: Supplementary file 1 — Data S1: joa370203‐sup‐0001‐Supinfo.docx. [file JOA3-41-e70203-s001.docx]

**Supplementary Data**

**Table S1**. Sensitivity analysis using alternative model or survival distribution assumptions.

**Table S2.** Sensitivity analysis to investigate if the effect of remote monitoring compliance on mortality or heart failure-related hospitalization was confounded by year of ICD/CRT implant.

**Figure X1**. Kaplan–Meier estimates of primary outcome, by the number of scheduled transmissions per year (≥2 vs. <2), when including only participants with available covariate data in the multivariable analysis.

**Figure X2**. Kaplan–Meier estimates of secondary outcomes, by the number of scheduled transmissions per year (≥2 vs. <2), when including only participants with available covariate data in the multivariable analysis. **A**. Mortality. **B**. Hospitalization for heart failure.

**Figure X3**. Remote monitoring compliance by year of ICD/CRT implantation.

**Table S1**. Sensitivity analysis using alternative model or survival distribution assumptions.

| **Outcome and model** | **Adjusted* hazard ratio (95% CI), ≥2 vs. <2 scheduled transmissions per year** | **P value** | **Adjusted*** **acceleration factor (95% CI), ≥2 vs. <2 scheduled transmissions per year** | **P value** |
| --- | --- | --- | --- | --- |
| **Mortality or HF-related hospitalization** |  |  |  |  |
| Cox PH | 0.26 (0.08 to 0.84) | 0.024 | – | – |
| AFT (log-normal) | – | – | 0.17 (0.05 to 0.57) | 0.004 |
| AFT (log-logistic) | – | – | 0.19 (0.05 to 0.77) | 0.020 |
| AFT (Weibull) | – | – | 0.20 (0.05 to 0.90) | 0.036 |
| **Mortality** |  |  |  |  |
| Cox PH | 0.42 (0.09 to 1.98) | 0.272 | – | – |
| AFT (log-normal) | – | – | 0.58 (0.26 to 1.28) | 0.177 |
| AFT (log-logistic) | – | – | 0.59 (0.25 to 1.39) | 0.228 |
| AFT (Weibull) | – | – | 0.60 (0.25 to 1.42) | 0.244 |
| **HF-related hospitalization** |  |  |  |  |
| Cox PH | 0.16 (0.02 to 1.13) | 0.066 | – | – |
| AFT (log-normal) | – | – | 0.06 (0.01 to 0.53) | 0.011 |
| AFT (log-logistic) | – | – | 0.06 (0.00 to 1.32) | 0.075 |
| AFT (Weibull) | – | – | 0.06 (0.00 to 1.65) | 0.098 |

*All models adjusted for age at ICD/CRT implantation, gender, baseline left ventricular ejection fraction prior to implantation, baseline diabetes, hypertension, prior myocardial infarction, and prior stroke or transient ischemic attack.

AFT=Accelerated failure time. CI=Confidence interval. HF=Heart failure. PH=Proportional hazards.

**Table S2**. Sensitivity analysis to investigate if the effect of remote monitoring compliance on mortality or heart failure-related hospitalization was confounded by year of ICD/CRT implant.

| **Outcome** | **Main analysis** | | **Sensitivity analysis** | |
| --- | --- | --- | --- | --- |
|  | **Adjusted*** **AF (95% CI), ≥2 vs. <2 scheduled transmissions per year** | **P value** | **Adjusted† AF (95% CI), ≥2 vs. <2 scheduled transmissions per year** | **P value** |
| Mortality or HF-related hospitalization | 0.24 (0.07 to 0.81) | 0.022 | 0.20 (0.06 to 0.68) | 0.009 |
| Mortality | 0.30 (0.07 to 1.34) | 0.115 | 0.27 (0.06 to 1.21) | 0.086 |
| HF-related hospitalization | 0.15 (0.02 to 1.15) | 0.068 | 0.15 (0.02 to 1.15) | 0.068 |

*In the main analysis, the multivariable model adjusted for age at ICD/CRT implantation, gender, baseline left ventricular ejection fraction prior to implantation, baseline diabetes, hypertension, prior myocardial infarction, and prior stroke or transient ischemic attack.

†In the sensitivity analysis, the multivariable model adjusted for all variables in the main analysis plus year of ICD/CRT implant (in or after 2016 vs. before 2016).

AF=Acceleration factor. CI=Confidence interval. HF=Heart failure.


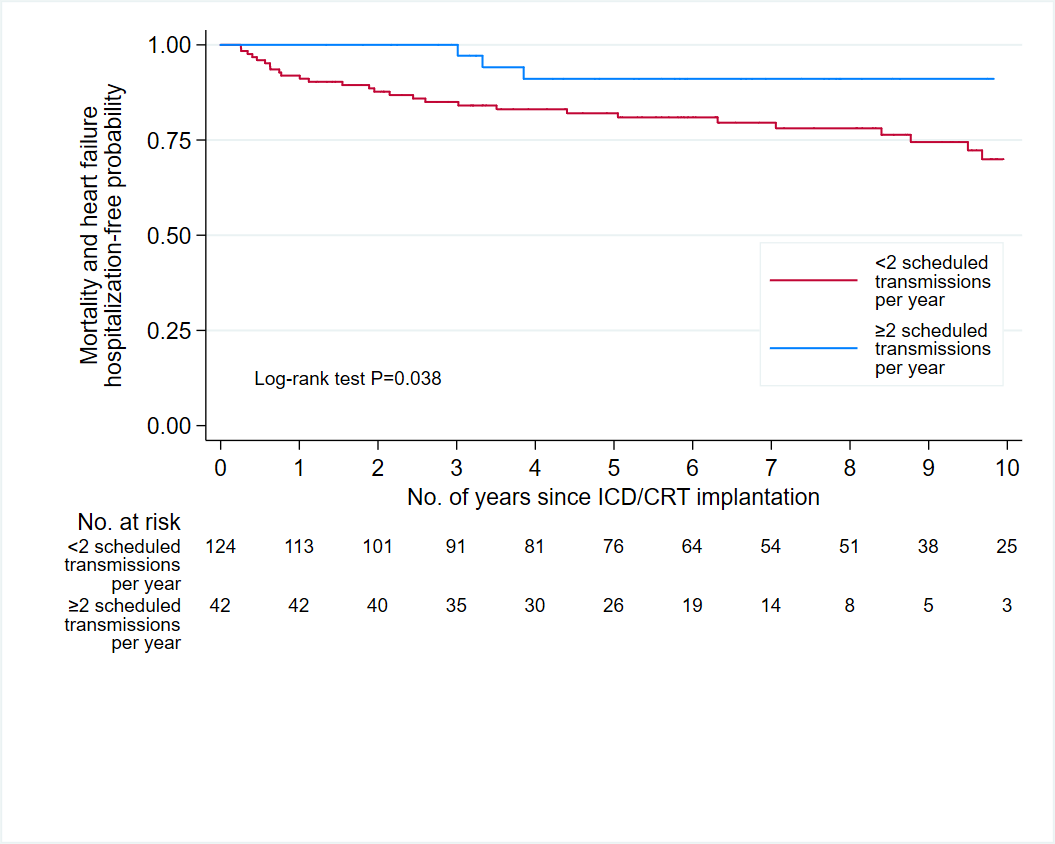


**Figure X1**. Kaplan–Meier estimates of primary outcome, by the number of scheduled transmissions per year (≥2 vs. <2), when including only participants with available covariate data in the multivariable analysis.

CRT=Cardiac resynchronization therapy. ICD=Implantable cardioverter defibrillator.

**
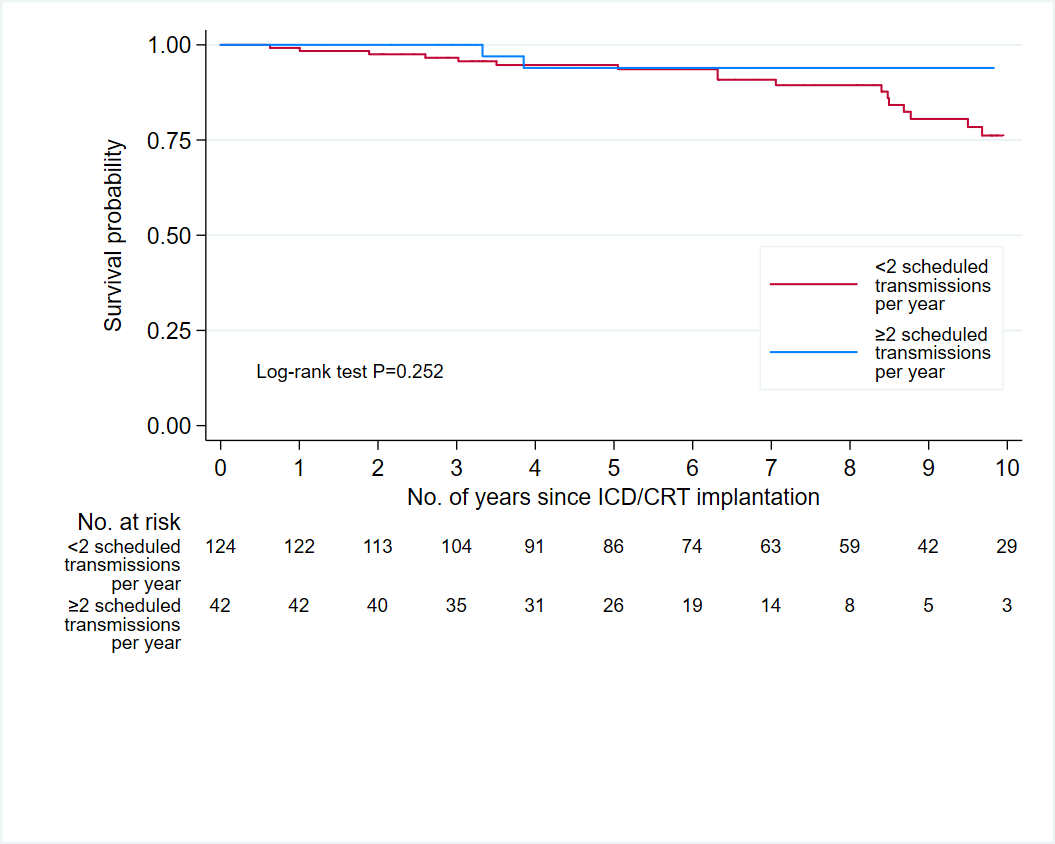
**

**A**


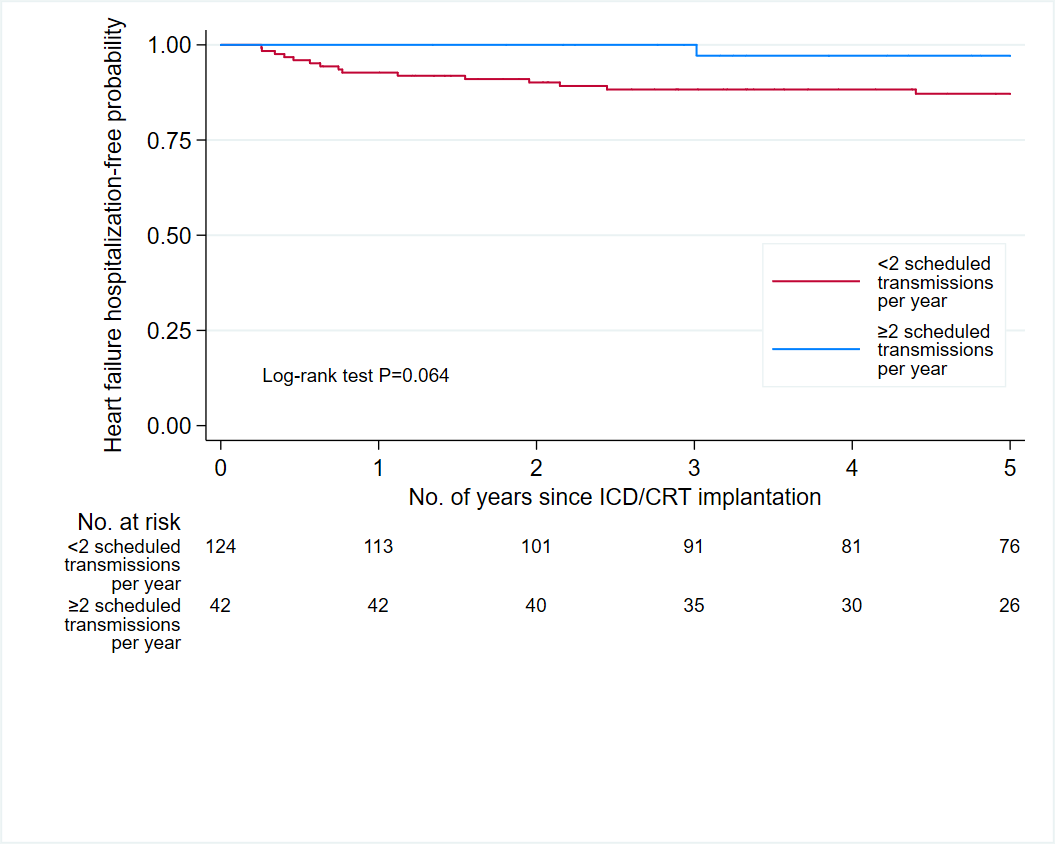


**B**

**Figure X2**. Kaplan–Meier estimates of secondary outcomes, by the number of scheduled transmissions per year (≥2 vs. <2), when including only participants with available covariate data in the multivariable analysis. **A**. Mortality. **B**. Hospitalization for heart failure.

CRT=Cardiac resynchronization therapy. ICD=Implantable cardioverter defibrillator.

**
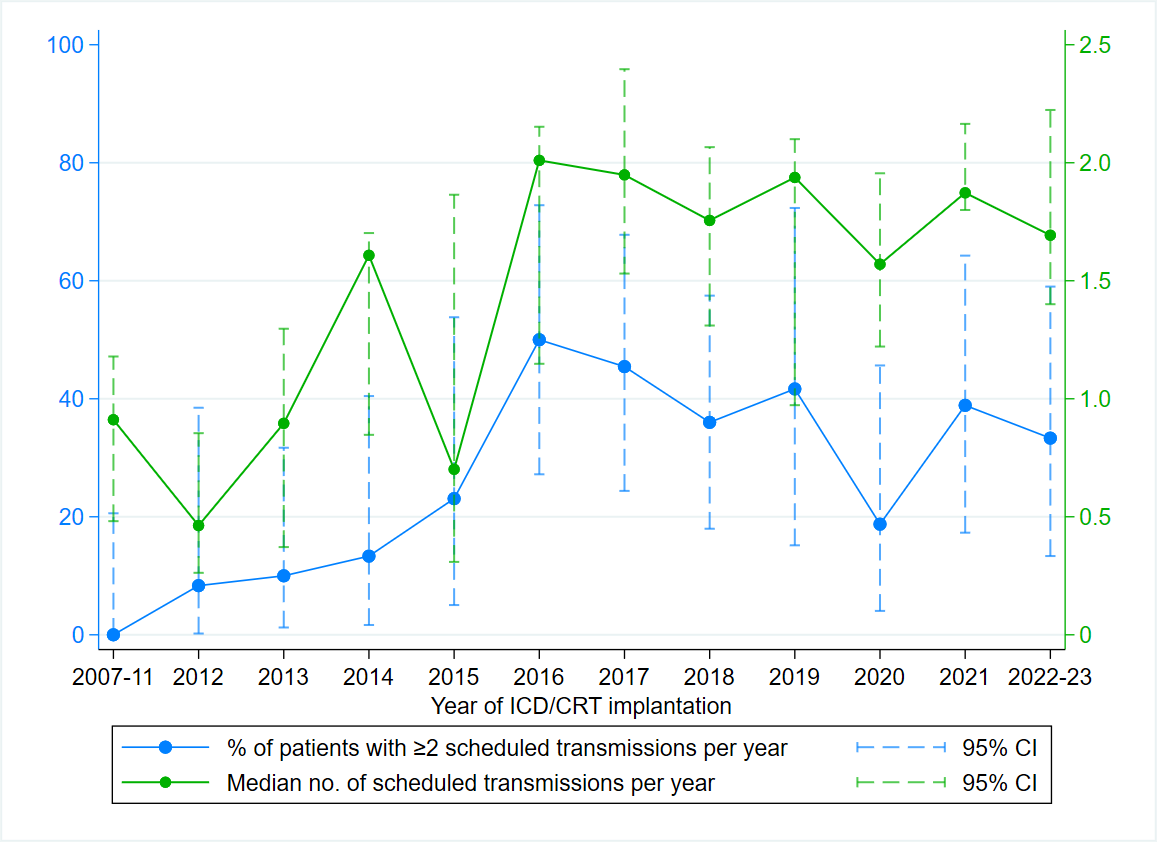
**

**Figure X3**. Remote monitoring compliance by year of ICD/CRT implantation.

CI=Confidence interval. CRT=Cardiac resynchronization therapy. ICD=Implantable cardioverter defibrillator.
